# Supplementary material for: Modeling glioblastoma heterogeneity as a dynamic network of cell states
Source: Mol Syst Biol. 2021 Sep 16;17(9):e10105. doi: 10.15252/msb.202010105 (PMC8444284; doi:10.15252/msb.202010105)
Supplement: Supplementary file 6 — Source Data for Figure 5 [file MSB-17-e10105-s004.zip › Figure5A_sourcedata/GSEA_3071/nosigdb_stateII.GseaPreranked.1622795151171/LARSSON_STATE_4.html]

Details for gene set LARSSON\_STATE\_4[GSEA]

|  || Dataset | state23071 |
| Phenotype | NoPhenotypeAvailable |
| Upregulated in class | na\_pos |
| GeneSet | LARSSON\_STATE\_4 |
| Enrichment Score (ES) | 0.8258098 |
| Normalized Enrichment Score (NES) | 4.932336 |
| Nominal p-value | 0.0 |
| FDR q-value | 0.0 |
| FWER p-Value | 0.0 |
Table: GSEA Results Summary

  

Fig 1: Enrichment plot: LARSSON\_STATE\_4      
 Profile of the Running ES Score & Positions of GeneSet Members on the Rank Ordered List

  

| PROBE | GENE SYMBOL | GENE\_TITLE | RANK IN GENE LIST | RANK METRIC SCORE | RUNNING ES | CORE ENRICHMENT || 1 | HIST1H1D |  |  | 0 | 2.323 | 0.0258 | Yes |
| 2 | CLSPN |  |  | 1 | 1.984 | 0.0479 | Yes |
| 3 | HIST1H4C |  |  | 2 | 1.902 | 0.0690 | Yes |
| 4 | RRM2 |  |  | 3 | 1.857 | 0.0896 | Yes |
| 5 | HIST1H1B |  |  | 7 | 1.669 | 0.1075 | Yes |
| 6 | TYMS |  |  | 8 | 1.665 | 0.1260 | Yes |
| 7 | MCM10 |  |  | 9 | 1.654 | 0.1443 | Yes |
| 8 | PCLAF |  |  | 10 | 1.638 | 0.1625 | Yes |
| 9 | CDK1 |  |  | 14 | 1.540 | 0.1789 | Yes |
| 10 | ZWINT |  |  | 16 | 1.510 | 0.1955 | Yes |
| 11 | UBE2C |  |  | 23 | 1.438 | 0.2099 | Yes |
| 12 | CENPU |  |  | 24 | 1.416 | 0.2257 | Yes |
| 13 | RAD51AP1 |  |  | 25 | 1.384 | 0.2411 | Yes |
| 14 | CENPK |  |  | 26 | 1.382 | 0.2564 | Yes |
| 15 | MYBL2 |  |  | 28 | 1.362 | 0.2713 | Yes |
| 16 | FBXO5 |  |  | 30 | 1.359 | 0.2862 | Yes |
| 17 | RRM1 |  |  | 37 | 1.310 | 0.2992 | Yes |
| 18 | ORC6 |  |  | 39 | 1.308 | 0.3135 | Yes |
| 19 | GINS2 |  |  | 40 | 1.306 | 0.3280 | Yes |
| 20 | TK1 |  |  | 42 | 1.281 | 0.3420 | Yes |
| 21 | MCM7 |  |  | 45 | 1.273 | 0.3557 | Yes |
| 22 | BRCA1 |  |  | 49 | 1.249 | 0.3688 | Yes |
| 23 | PCNA |  |  | 58 | 1.214 | 0.3803 | Yes |
| 24 | CDC45 |  |  | 59 | 1.212 | 0.3938 | Yes |
| 25 | HIST1H1C |  |  | 60 | 1.206 | 0.4072 | Yes |
| 26 | MAD2L1 |  |  | 61 | 1.199 | 0.4205 | Yes |
| 27 | UBE2T |  |  | 62 | 1.192 | 0.4338 | Yes |
| 28 | ATAD2 |  |  | 63 | 1.189 | 0.4470 | Yes |
| 29 | TOP2A |  |  | 72 | 1.132 | 0.4576 | Yes |
| 30 | MCM5 |  |  | 74 | 1.128 | 0.4699 | Yes |
| 31 | UHRF1 |  |  | 79 | 1.113 | 0.4813 | Yes |
| 32 | MCM4 |  |  | 81 | 1.110 | 0.4933 | Yes |
| 33 | GMNN |  |  | 83 | 1.108 | 0.5054 | Yes |
| 34 | HIST1H1E |  |  | 90 | 1.088 | 0.5160 | Yes |
| 35 | TMPO |  |  | 99 | 1.056 | 0.5258 | Yes |
| 36 | NCAPG |  |  | 100 | 1.056 | 0.5375 | Yes |
| 37 | DHFR |  |  | 101 | 1.051 | 0.5492 | Yes |
| 38 | MND1 |  |  | 104 | 1.036 | 0.5602 | Yes |
| 39 | MCM3 |  |  | 105 | 1.035 | 0.5717 | Yes |
| 40 | MKI67 |  |  | 107 | 1.031 | 0.5829 | Yes |
| 41 | TPX2 |  |  | 111 | 1.025 | 0.5936 | Yes |
| 42 | RFC2 |  |  | 112 | 1.022 | 0.6049 | Yes |
| 43 | DUT |  |  | 118 | 1.009 | 0.6149 | Yes |
| 44 | ASF1B |  |  | 120 | 1.007 | 0.6258 | Yes |
| 45 | SMC2 |  |  | 123 | 1.005 | 0.6365 | Yes |
| 46 | CHAF1A |  |  | 127 | 0.999 | 0.6469 | Yes |
| 47 | CKS1B |  |  | 128 | 0.999 | 0.6580 | Yes |
| 48 | FANCI |  |  | 138 | 0.984 | 0.6667 | Yes |
| 49 | ESCO2 |  |  | 144 | 0.971 | 0.6762 | Yes |
| 50 | RANBP1 |  |  | 149 | 0.958 | 0.6859 | Yes |
| 51 | E2F1 |  |  | 153 | 0.951 | 0.6957 | Yes |
| 52 | GTSE1 |  |  | 167 | 0.929 | 0.7028 | Yes |
| 53 | CENPM |  |  | 178 | 0.901 | 0.7103 | Yes |
| 54 | H2AFZ |  |  | 213 | 0.864 | 0.7114 | Yes |
| 55 | PBK |  |  | 214 | 0.864 | 0.7210 | Yes |
| 56 | CENPH |  |  | 221 | 0.855 | 0.7290 | Yes |
| 57 | EZH2 |  |  | 233 | 0.839 | 0.7356 | Yes |
| 58 | DBF4 |  |  | 240 | 0.832 | 0.7434 | Yes |
| 59 | LMNB1 |  |  | 251 | 0.824 | 0.7500 | Yes |
| 60 | HMGB2 |  |  | 276 | 0.780 | 0.7527 | Yes |
| 61 | SMC4 |  |  | 279 | 0.777 | 0.7608 | Yes |
| 62 | DEK |  |  | 302 | 0.754 | 0.7637 | Yes |
| 63 | KIF4A |  |  | 309 | 0.746 | 0.7705 | Yes |
| 64 | CKS2 |  |  | 312 | 0.745 | 0.7783 | Yes |
| 65 | ALYREF |  |  | 315 | 0.741 | 0.7860 | Yes |
| 66 | CENPN |  |  | 320 | 0.737 | 0.7932 | Yes |
| 67 | ANP32B |  |  | 325 | 0.734 | 0.8004 | Yes |
| 68 | DTYMK |  |  | 364 | 0.701 | 0.7987 | Yes |
| 69 | SIVA1 |  |  | 380 | 0.690 | 0.8027 | Yes |
| 70 | KIF15 |  |  | 404 | 0.676 | 0.8044 | Yes |
| 71 | SGO2 |  |  | 415 | 0.670 | 0.8094 | Yes |
| 72 | KPNA2 |  |  | 420 | 0.669 | 0.8158 | Yes |
| 73 | RAN |  |  | 424 | 0.667 | 0.8225 | Yes |
| 74 | BIRC5 |  |  | 505 | 0.628 | 0.8095 | Yes |
| 75 | SNRPD1 |  |  | 523 | 0.615 | 0.8121 | Yes |
| 76 | SGO1 |  |  | 528 | 0.612 | 0.8179 | Yes |
| 77 | RPA3 |  |  | 532 | 0.611 | 0.8240 | Yes |
| 78 | CENPF |  |  | 575 | 0.588 | 0.8200 | Yes |
| 79 | CCNB1 |  |  | 579 | 0.587 | 0.8258 | Yes |
| 80 | NUSAP1 |  |  | 654 | 0.550 | 0.8135 | No |
| 81 | H2AFX |  |  | 671 | 0.544 | 0.8155 | No |
| 82 | TUBB |  |  | 678 | 0.538 | 0.8200 | No |
| 83 | PHF19 |  |  | 745 | 0.518 | 0.8093 | No |
| 84 | TUBA1B |  |  | 862 | 0.481 | 0.7857 | No |
| 85 | HNRNPD |  |  | 1177 | 0.401 | 0.7119 | No |
| 86 | PTMA |  |  | 1233 | 0.390 | 0.7025 | No |
| 87 | STMN1 |  |  | 1249 | 0.387 | 0.7031 | No |
| 88 | LSM4 |  |  | 1676 | 0.313 | 0.6003 | No |
| 89 | HMGB1 |  |  | 1720 | 0.308 | 0.5930 | No |
Table: GSEA details [plain text format]

  

Fig 2: LARSSON\_STATE\_4: Random ES distribution      
 Gene set null distribution of ES for **LARSSON\_STATE\_4**
